# Ginsenoside Rb1 affects mitochondrial Ca^2+^ transport and inhibits fat deposition and fibrosis by regulating the wnt signaling pathway to treat rotator cuff tears via docking with SFRP1

**DOI:** 10.1186/s10020-024-01009-0

**Published:** 2024-12-02

**Authors:** Yuesong Yin, Hai Hu, Yian Yang, Song Wu

**Affiliations:** 1 Orthopedics Department, The Third Xiangya Hospital of Central South University, No.138 Tongzipo Road, Yuelu District, Changsha, 410013 China; 2 Oncology Department, The Third Xiangya Hospital of Central South University, Changsha, 410013 China

**Keywords:** Ginsenoside Rb1, SFRP1, Wnt, Ca^2+^, Rotator cuff tears

## Abstract

**Background:**

Rotator cuff tears (RCTs) are among the most common musculoskeletal disorders that affect quality of life. This study aimed to investigate the efficacy of ginsenoside Rb1 in RCTs and the mechanisms involved.

**Methods:**

First, a fibrotic model of FAPs was induced, and FAPs were cultured in media supplemented with different concentrations of ginsenoside Rb1. Next, a rat model of RCTs was constructed and treated with ginsenoside Rb1. Molecular docking was subsequently utilized to detect the binding of ginsenoside Rb1 and SFRP1. Finally, SFRP1 was knocked down and overexpressed in vivo and in vitro to investigate the mechanism of ginsenoside Rb1 and SFRP1 in RCTs.

**Results:**

Compared with the Normal group, FAP viability was decreased, but Collagen II, FN and α-SMA levels were increased in the Control group. After treatment with different concentrations of ginsenoside Rb1, FAP viability increased, but Collagen II, FN and α-SMA levels decreased. Among them, 60 µM ginsenoside Rb1 had the best effect. In vivo experiments revealed that ginsenoside Rb1 improved RCTs in rats. Molecular docking revealed the binding of ginsenoside Rb1 to SFRP1. Additionally, SFRP1 levels were lower in the Control group than in the Normal group. After treatment with ginsenoside Rb1, SFRP1 levels increased. In vivo, overexpressing SFRP1 along with ginsenoside Rb1 treatment further alleviated tendon tissue fibroblast infiltration and fat accumulation and further reduced the expression of Collagen II, FN, and α-SMA. In vitro, overexpressing SFRP1 along with ginsenoside Rb1 treatment further decreased the expression of CaMKII, PLC, PKC, Wnt, and β-catenin, further decreased the Ca^2+^ fluorescence intensity and mitochondrial length, increased the red/green intensity, and decreased the MitoSOX fluorescence intensity. Additionally, overexpressing SFRP1 along with ginsenoside Rb1 treatment further increased cell proliferation, decreased apoptosis, reduced the protein expression of Collagen II, FN, and α-SMA in muscle tissue, and further reduced the levels of TNF-α, IL-1β, and IL-6 in the cell supernatant.

**Conclusions:**

Ginsenoside Rb1 inhibited the activation of the Wnt signaling pathway by promoting SFRP1 expression, thereby inhibiting mitochondrial function and Ca^2+^ absorption to treat fat infiltration and muscle fibrosis caused by RCTs.

**Supplementary Information:**

The online version contains supplementary material available at 10.1186/s10020-024-01009-0.

## Introduction

Rotator cuff tears (RCTs), some of the most common musculoskeletal disorders, are caused by degeneration or damage to the shoulder tendons at the anatomical neck of the humerus head, often resulting in repeated shoulder pain and limited movement (Chen et al. 2024; Zhou et al. 2023). The incidence of RCTs increases with age. In people over 75 years of age, the rate of asymptomatic full-layer tears is 40% (Meng et al. 2022). The pathophysiology of RCTs is complex and includes interactions between tendons, bones and muscles (Bedi et al. 2024). Metabolic factors such as diabetes, thyroid disease, high cholesterol, vitamin D deficiency, obesity, and smoking are linked to a greater risk of RCTs (Yoon et al. 2023). Early diagnosis of RCTs is essential for appropriate and timely treatment (Iio et al. 2024). However, the poor self-repair ability of the tendon and the limitations of current surgical and injection therapies still represent major drawbacks (Chen et al. 2020). Hence, delving more deeply into the mechanism of RCTs is crucial.

Ginsenoside Rb1, a protopanaxanol saponin, is the main bioactive component in ginseng. As a nervous system-protective drug with antioxidant, antiapoptotic and anti-inflammatory effects, ginsenoside Rb1 has received increasing attention (Gong et al. 2022). Ginsenoside Rb1 exerts these effects by modulating mitochondrial energy metabolism, mitochondrial division and fusion, apoptosis, oxidative stress, reactive oxygen species (ROS) release, mitochondrial autophagy, and the mitochondrial membrane potential. Consequently, mitochondria represent crucial targets of ginsenoside Rb1 (Zhou et al. 2019). Ginsenoside Rb1 can reduce the energy charge of cells and improve mitochondrial dysfunction, thereby increasing cardiac resistance to ischemia/reperfusion injury (Li et al. 2017; Yan et al. 2014). In addition, ginsenoside Rb1 was able to improve renal fibrosis induced by bavacin (Ni et al. 2022a, b). Therefore, we speculate that ginsenoside Rb1 may alleviate RCTs-induced muscle fibrosis, but the specific pathway responsible for its effect remains to be explored.

The inflammatory response has been implicated in the development of fatty degeneration after rotator cuff injuries (Oak et al. 2014). Studies have shown that the activation of secreted frizzled-related protein 1 (SFRP1) can repress the Wnt/Ca^2+^ signaling pathway (Nachtigall et al. 2023) and prevent Ca^2+^ overload and subsequent death in damaged cells (Fuping et al. 2019). In addition, SFRP1 inhibits the Wnt/β-catenin signaling pathway. SFRP1 overexpression alleviates myocardial fibrosis by repressing the Wnt/β-catenin signaling pathway (Liu et al. 2021). However, no studies have investigated the regulatory relationship between ginsenoside Rb1 and SFRP1. We found that ginsenoside Rb1 and SFRP1 could interact through molecular docking. Therefore, we speculate that ginsenoside Rb1 may treat RCTs by regulating SFRP1.

Based on these findings, we hypothesize that ginsenoside Rb1 inhibits the activation of the Wnt signaling pathway by docking with SFRP1, thereby preventing Ca^2+^ overload in damaged cells and thus treating fat infiltration and muscle fibrosis caused by RCTs. To this end, we constructed RCTs model, treated the animals with ginsenosideRb1, and observed the therapeutic effect of ginsenoside Rb1 on fat infiltration and muscle fibrosis. In addition, we extracted interstitial fibrous adipocyte progenitors (FAPs) and observed the mitochondrial Ca^2+^ transport mode by constructing a cellular fibrosis model and knocking down SFRP1 to explore the progress of alleviating muscle fibrosis. Our study may provide new ideas for treating RCTs.

## Materials and methods

### Animals

The study was approved by the Animal Welfare Committee of Central South University (No. 2019sydw0198). Female SD rats (12 weeks) were purchased from Hunan SJA Laboratory Animal Co., Ltd. Formal experimental operations were scheduled after one week of adaptive feeding. Experiment 1: The rats were divided into the Sham, RCTs, and Rb1 groups, with 8 rats in each group. 3% sodium pentobarbital (80 mg/kg) was injected intraperitoneally for anesthesia. The right shoulder of each rat was shaved and disinfected to keep it sterile. A rotator cuff incision was made. The scapula was palpated, and a 3 cm longitudinal incision was made. The deltoid was directly separated, and the supraspinatus tendon was identified. Using anatomical scissors, the supraspinatus tendon was dissected, and the tendon at the insertion point was cut with a blade. Subsequently, large nodules were significantly exposed, and two bone tunnels were drilled in parallel (Yoon et al. 2022). In the Sham group, only an incision was made without separation of the deltoid muscle, and suturing was performed after the incision. After deltoid separation, the rats in group Rb1 were intraperitoneally injected with 60 mg/kg/d ginsenoside Rb1 (HY-N0039, MedChemExpress) for 4 weeks. Experiment 2: The rats were divided into the Sham, RCTs, Rb1, Rb1 + oe-NC, Rb1 + oe-SFRP1, Rb1 + si-NC, and Rb1 + si-SFRP1 groups, with 8 rats in each group. Rats in the Rb1 + oe-NC, Rb1 + oe-SFRP1, Rb1 + si-NC, and Rb1 + si-SFRP1 groups were also injected with the oe-NC, oe-SFRP1, si-NC, or si-SFRP1 virus for 4 weeks after the injection of Rb1. After four weeks of treatment, the rats were euthanized, and tendon tissue was harvested. All the viruses were purchased from Honorgene.

### Extraction and identification of FAPs

The muscles were harvested from the hind limbs of the SD rats. Nonmuscle tissue was meticulously removed. The trimmed muscles were minced, digested with 0.2% Collagenase II, and incubated at 37 °C for 1 h. Muscle slurries were then filtered through 100 μm and 40 μm cell strainers (431752 and 431750, BD Bioscience). After the red blood cells were removed, the cells were resuspended in PBS wash buffer containing 2% FBS (Kang et al. 2018). Freshly sorted FAPs were cultured in F10 medium supplemented with 20% FBS and 1% penicillin/streptomycin. The cells were passaged twice before being treated with terminal differentiation medium for 2 weeks. No substantial proliferation was observed after the cells were switched to differentiation medium. The cells were then fixed with 4% paraformaldehyde and identified by staining for alpha-smooth muscle actin (α-SMA) (Davies et al. 2022).

### Cell culture and treatment

FAPs were cultured in F10 medium supplemented with 20% FBS and 1% penicillin/streptomycin at 37 °C in a 5% CO_2_ environment with saturated humidity in an incubator. The logarithmically growing FAPs were seeded into a six-well plate, and after the cells were attached to the wall, they were processed into the following groups: Normal (FAPs cultured normally), Control (FAPs with induced fibrosis), Rb1 10 µM (FAPs with induced fibrosis were cultured in medium containing 10 µM ginsenoside Rb1), Rb1 20 µM (FAPs with induced fibrosis were cultured in medium containing 20 µM ginsenoside Rb1), Rb1 40 µM (FAPs with induced fibrosis were cultured in medium containing 40 µM ginsenoside Rb1), Rb1 60 µM (FAPs with induced fibrosis were cultured in medium containing 60 µM ginsenoside Rb1), and Rb1 100 µM (FAPs with induced fibrosis were cultured in medium containing 100 µM ginsenoside Rb1). The optimal concentration of ginsenoside Rb1 was 60 µM, and the cells were further divided into the following groups: Normal (FAPs cultured normally), Control (FAPs with induced fibrosis), and Rb1 60 µM (FAPs with induced fibrosis were cultured in medium containing 60 µM ginsenoside Rb1).

In addition, SFRP1 was knocked down and overexpressed in cells, which was further divided into the following groups: Control (FAPs with induced fibrosis), Rb1 (FAPs with induced fibrosis were cultured in media supplemented with 60 µM ginsenoside Rb1), Rb1 + oe-NC group (FAPs with induced fibrosis were transfected with oe-NC and cultured in media supplemented with 60 µM ginsenoside Rb1), Rb1 + oe-SFRP1 (FAPs with induced fibrosis were transfected with oe-SFRP1 and cultured in media supplemented with 60 µM ginsenoside Rb1), Rb1 + si-NC group (FAPs with induced fibrosis were transfected with si-NC and cultured in media supplemented with 60 µM ginsenoside Rb1), and Rb1 + si-SFRP1 (FAPs with induced fibrosis were transfected with si-SFRP1 and cultured in media supplemented with 60 µM ginsenoside Rb1). All the plasmids used for transfection were purchased from Honorgene. Transfection was performed using Lip 2000 (11668019, Invitrogen) according to the manufacturer’s instructions.

### Cell counting kit 8 (CCK-8) assay

Cell proliferation was assessed using the CCK-8 assay. The cells from the aforementioned groups were harvested, counted, and then seeded into a 24-well plate at a density of 5 × 10^3^ cells per well with 300 µL of media per well. Three wells were set up for each group, and after culture and adhesion, 30 µL/well of CCK-8 (NU679, DOJINDO) was added to each well for the corresponding time after treatment, as described above. After an incubation at 37 °C with 5% CO_2_ for 4 h, the absorbance at 450 nm was measured with a microplate reader.

### Quantitative real-time PCR (qRT‒PCR)

The expression levels of Collagen II, FN, α-SMA, SFRP1 and CaMK II were detected via qRT‒PCR. First, a TRIzol total RNA extraction kit (15596026, Thermo) was used to extract total RNA, and the concentration and purity were determined. Then, an mRNA reverse transcription kit (CW2569, CWBIO, China) was used to reverse transcribe the mRNA into cDNA. An Ultra SYBR mixture (CW2601, CWBIO, China) was used to examine gene expression on an ABI 7900 system. The following reaction conditions were used: predenaturation at 95 °C for 10 min, 40 cycles of denaturation at 94 °C for 15 s, and annealing at 60 °C for 30 s. Gene expression was determined using the 2^−ΔΔCt^ method, with β-actin serving as an internal reference gene. The primers used are as follows: Collagen II-F—TTACCCTGGCAACATTGGTC, Collagen II-R—CCTTGTCACCTCGAATACCTTG; FN-F—CGCCCTTTTCTCCTGTTGTGG, FN-R—CTCCTTCCTCGCTCAGTTCGT; α-SMA-F—GGATCAGCGCCTTCAGTTCT, α-SMA-R—AGGGCTAGAAGGGTAGCACA; SFRP1-F—TCGAAGCCCCAAGGTACAAC, SFRP1-R—GTTCGATGATGGCCTCCGAT; CaMK II-F—CCTGAACCCTCACATCCACC, CaMK II-R—CTGGCCTGGTCCTTCAATGG; Pparγ-F—CCACACTATGAAGACATCCCGTT, Pparγ-R—CAGGCTCTACTTTGATCGCACT; C/ebpα-F—CCTCCGTCCCTGTCCTTAGA, C/ebpα-R—AAGCAAGGGGCTAAGAACCC; Fas-F—GTGTGGTAGGCTTGGTGAACTGTC, Fas-R—GTGAGATGTGCTGCTGAGGTTGG; Fabp4-F—AAGCTGGTGGTGGAATGTGT, Fabp4-R—ATTTCAGTCCAGGGCCTCGT; Apn-F—GCTCTTTGTTTGGAGAGGGGA, Apn-R—GGCGGTGACACTAATCTTCCT; β-actin-F—ACAGCAACAGGGTGGTGGAC, and β-actin-R—TTTGAGGGTGCAGCGAACTT.

### Western blot

Western blot was performed to detect the Collagen II, FN, α-SMA, SFRP1, CaMK II, Caspase-3, Bax, and Bcl-2 levels. First, the samples were treated with RIPA buffer (AWB0136, Abiowell) to extract total protein. The proteins were then separated by SDS‒PAGE and transferred onto a nitrocellulose membrane. The membrane was subsequently blocked with 5% skim milk. Next, the membrane was incubated with primary antibodies overnight at 4 °C. The primary antibodies used included Collagen II (AWA01318, 1:1000, Abiowell), FN (AWA00143, 1:1000, Abiowell), α-SMA (AWA00774, 1:1000, Abiowell), SFRP1 (AWA01318, 1:1000, Abiowell), CaMKII (AWA51690, 1:1000, Abiowell), Caspase-3 (AWA45560, 1:1000, Abiowell), Bax (AWA00856, 1:1000, Abiowell), Bcl-2 (AWA00387, 1:1000, Abiowell), Pparγ (16643-1-AP, 1:1000, Proteintech), C/ebpα (29388-1-AP, 1:1000, Proteintech), Fas (ab271016, 1:1000, Abcam), Fabp4 (12802-1-AP, 1:5000, Proteintech), Apn (14553-1-AP, 1:1000, Proteintech), and β-actin (AWA80002, 1:5000, Abiowell). The membranes were incubated with HRP-conjugated goat anti-mouse IgG (SA00001-1, 1:5000, Proteintech) or HRP-conjugated goat anti-rabbit IgG (SA00001-2, 1:6000, Proteintech) at room temperature for 1.5 h. Finally, the membranes were incubated with enhanced chemiluminescence (ECL) reagent (AWB0005, Abiowell) for 1 min and placed on an imaging system for analysis. Protein levels were analyzed using Quantity One 4.6.6 software, with β-actin serving as an internal reference.

### Hematoxylin‒eosin (HE) staining

HE staining was used to assess damage to rat tendon tissues. The tissue sections were first incubated at 60 °C for 12 h and then dewaxed in water. The sections were deparaffinized by immersion in xylene three times for 20 min each. The sections were subsequently transferred through ethanol solutions of various concentrations (100%, 100%, 95%, 85%, and 75%) for 5 min at each stage. The sections were then stained with hematoxylin for 1–10 min and blued with PBS. Eosin staining was then performed for 1–5 min, after which the samples were subsequently rinsed with distilled water. The sections were dehydrated using a series of alcohol solutions (95–100%) for 5 min at each stage or, alternatively, allowed to air dry. Next, the sections were transferred to xylene twice for 10 min each. Finally, the sections were mounted with neutral gum for microscopic observation.

### Masson staining

Masson staining kit (AWI0253a, Abiowell) was used to assess fibrosis in rat tendon tissue. The sections were baked and deparaffinized. A suitable amount of nuclear staining solution was carefully added dropwise to cover the entire sample, which was subsequently stained for 10 s. PBS was used for bluing. An appropriate amount of cytoplasmic staining solution was added to cover the whole tissue, which was subsequently stained for 5 min. The sections were treated with color separation solution for approximately 30 s to achieve color separation, after which the solution was discarded. The sections were then stained for approximately 30 s by adding an adequate amount of counterstaining solution to cover the entire tissue. The sections were rinsed well with anhydrous ethanol and made transparent with xylene. After being sealed with neutral gum, the sections were observed under a light microscope (BA210T, Motic).

### Oil red O staining

The lipid distribution in rat tendon tissues was detected via oil red O staining (Vargas-Vila et al. 2022). The samples were coated with OTC compound for embedding, frozen, and cut at a thickness of was 6–8 μm. One hundred microliters of oil red O dye solution was added to sections from each sample, which were placed in a humid chamber and incubated at room temperature for 20–40 min. The dye solution was discarded. The sections were differentiated in 70% alcohol until the grains were clear, and the differentiation was stopped with distilled water. Hematoxylin was used to stain the sections for 1–3 min, and PBS was used to turn them blue. Buffered glycerol was used to seal the sections, which were then observed under a microscope. The lipid droplets were red, and the nuclei were blue.

### Immunofluorescence (IF) staining

The expression of SFRP1, Collagen II, fibronectin (FN), and α-SMA in rat tendon tissues was evaluated via IF staining. The tissue sections were incubated at 60 °C for 12 h, dewaxed in water, and subjected to antigen retrieval by heating. The sections were subsequently immersed in a sodium borohydride solution at room temperature for 30 min. Then, the sections were briefly soaked in a 75% ethanol solution for 15 s to 1 min and immersed in Sudan black dye solution at room temperature for 15 min. The sections were blocked with 10% normal serum and 5% BSA for 1 h. Appropriately diluted primary antibodies against SFRP1 (26460-1-AP, 1:100, Proteintech), Collagen II (ab34712, 1:100, Abcam), FN (ab268020, 1:100, Abcam), and α-SMA (BM0002, 1:300, BOSTER) were incubated with the sections at 4 °C overnight. The sections were then incubated with the secondary antibody at 37 °C for 30 min and washed with PBS. DAPI working solution was applied to stain the nuclei at 37 °C for 10‒20 min. The sections were then mounted with buffered glycerin, protected from light, and observed under a fluorescence microscope.

### Molecular docking

Molecular docking was utilized to confirm the binding between ginsenoside Rb1 and SFRP1. AutoDock VINA 1.1.2 software was employed to perform docking simulations of the compounds and proteins, utilizing semiempirical free energy fields to predict receptor‒ligand binding energies (Jiang et al. 2021). A subsequent chemical analysis using PyMOL revealed that the compound effectively bound within the protein cavity and interacted with the surrounding amino acids.

### Drug affinity responsive target stability (DARTS) assay

In accordance with previous research (Schulte et al. 2018), a DARTS assay was performed on SFRP1 in FAP lysates. The lysates were exposed to different concentrations of ginsenoside Rb1 (1–100 µM) at room temperature for 35–45 min. Then, the lysates were incubated with thermolysin (HY-P1748, MCE, 1:100 and 1:200 total enzymes to total substrate ratios) at room temperature for 30 min. SFRP1 levels were measured by Western blot.

### Immunocytochemistry (ICC)

ICC was used to detect β-catenin expression in FAPs. The slices were fixed with 4% paraformaldehyde. They were then permeabilized by adding 0.3% Triton X-100 and incubated at 37 °C for 30 min. Endogenous enzymes were deactivated by adding 3% H_2_O_2_, and the mixture was allowed to react for 10 min at room temperature. The primary antibody against β-catenin (17565-1-AP, 1:100, Proteintech) was incubated with the slices at 4 °C overnight. Subsequently, 50–100 µL of anti-rabbit IgG antibody conjugated with HRP was incubated with the sections at 37 °C for 30 min. DAB working solution was added dropwise and incubated at room temperature for 1–5 min, and the reaction time was monitored under a microscope. Hematoxylin was added, and the sections were restained for 5–10 min. The sections were then rinsed with distilled water, followed by a bluing step with PBS. The sections were subsequently dehydrated in a series of alcohol solutions (ranging from 60 to 100%), with each step lasting 5 min. The slices were removed, immersed in xylene twice for 10 min each, sealed with neutral gum, and then observed under a microscope.

### Fluorescent calcium imaging

Changes in Ca^2+^ absorption were detected via fluorescence calcium imaging. The 2 mM Fluo-3 AM storage solution (S1056, Beyotime) was added to the cell culture at a dilution of 1:1000, resulting in a final concentration of 2 µM. The cell culture medium was discarded, AM dyeing working medium prewarmed at 37 °C was added, and the cells were incubated at 37 °C for 15 min. The AM dyeing solution was discarded, and fresh cell culture medium was added. The cells were then visualized under a fluorescence microscope, and images were captured.

### MitoTracker Red CMXRos staining

MitoTracker Red CMXRos (C1049B, Beyotime) was utilized to detect mitochondrial morphology. MitoTracker Red CMXRos storage solution (1 mM) was added to the cell culture medium at a ratio of 1:500, resulting in a final concentration of 200 nM. The cell culture medium was discarded, MitoTracker Red CMXRos staining working medium prewarmed at 37 °C was added, and the cells were incubated at 37 °C for 15 min. MitoTracker Red CMXRos staining solution was discarded, and prewarmed 10 µg/ml Hoechst was added and incubated for 15 min at 37 °C. The Hoechst staining solution was discarded, and fresh cell culture medium was added. The cells were subsequently observed under a fluorescence microscope, and images were captured.

### JC-1 detection

The mitochondrial membrane potential was assessed using a JC-1 kit (C2006, Beyotime). The cells were collected and resuspended in 0.5 mL of cell culture medium containing serum and phenol red. JC-1 dye solution (0.5 mL) was added, mixed well, and then incubated at 37 °C for 20 min. JC-1 staining buffer (1×) was prepared by diluting 1 mL of JC-1 staining buffer (5×) with 4 mL of distilled water and keeping it on ice. After the incubation period, the cells were centrifuged at 600 × g for 5 min at 4 °C, after which the cell pellets were collected. The supernatant was carefully removed, and the cell pellets were washed twice with JC-1 staining buffer (1×). Following resuspension in 100 µL of JC-1 staining buffer (1×), a flow cytometry analysis was performed.

### MitoSOX detection

MitoSOX (M36005, Gibco) was used to detect ROS production in the mitochondria. Thirteen microliters of DMSO was added to fully dissolve MitoSOX in 1 mM mother liquor, and the dye liquor was diluted with basal culture medium (the mother liquor was 1 mM, and the working concentration was 200 nM). The cell culture medium was removed, the MitoSOX staining working solution prewarmed at 37 °C was added, and the cells were incubated at 37 °C for 15 min. The MitoSOX working solution was removed, and 10 µg/ml Hoechst was added and incubated at 37 °C for 15 min. The Hoechst staining solution was discarded, and fresh cell culture medium was added. The cells were subsequently observed under a fluorescence microscope, and images were captured.

### Cell apoptosis

The cells were harvested by digestion with EDTA-free trypsin, followed by centrifugation at 2000 rpm to collect approximately 3.2 × 10^5^ cells. Subsequently, 500 µL of binding buffer, 5 µL of Annexin V-APC (KGA1030, KeyGEN BioTECH), and 5 µL of propidium iodide were added. The reaction was performed in the dark. Within 1 h, the cells were analyzed using flow cytometry.

### Enzyme-linked immunosorbent assay (ELISA)

ELISAs were conducted to measure the TNF-α, IL-1β, and IL-6 levels in the cell supernatant. For this purpose, TNF-α (CSB-E11987r, CUSABIO), IL-1β (CSB-E08055r, CUSABIO), and IL-6 (CSB-E04640r, CUSABIO) quantitative ELISA kits were utilized according to the provided instructions.

### Statistical analysis

The statistical analysis was conducted with GraphPad Prism 8.0 software. Differences between two or more groups were assessed using Student’s t test or one-way analysis of variance (ANOVA). The measurement data are presented as the means ± standard deviations. *P* < 0.05 indicated statistical significance.

## Results

### Screening of the optimal ginsenoside Rb1 concentration

**Fig. 1:**
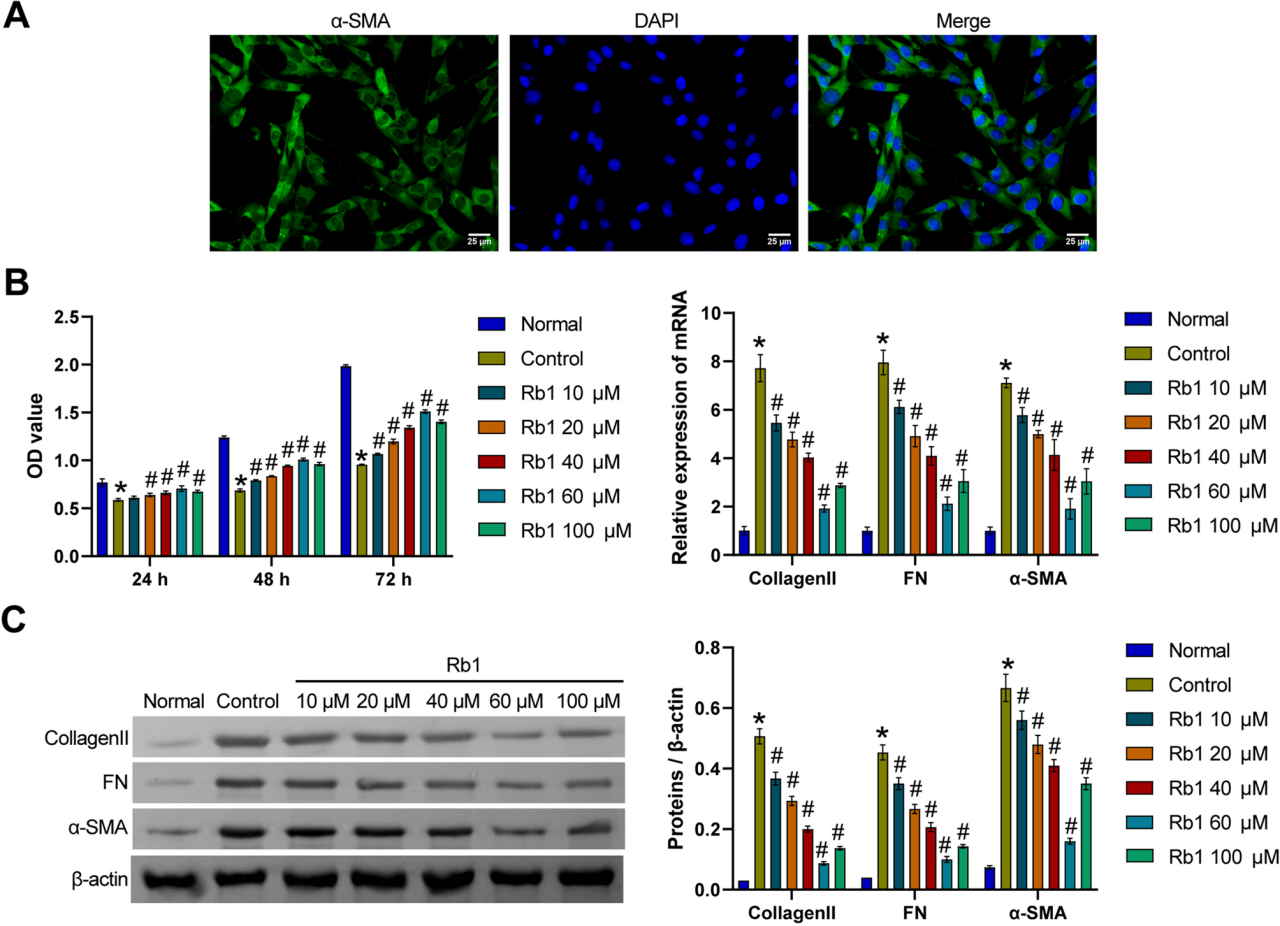
Screening of the optimal ginsenoside Rb1 concentration. **A**. IF staining for α-SMA. **B**. Cell proliferation was assessed using the CCK-8 assay. **C** and **D**. The levels of the Collagen II, FN and α-SMA mRNAs and proteins were detected via qRT‒PCR and Western blot. *P < 0.05 compared with the Normal group and #*P* < 0.05 compared with the Control group

First, we extracted and identified FAPs. Figure 1A showed that we successfully identified FAPs. Then, we induced a fibrotic model of FAPs and treated them with F10 medium containing different concentrations of ginsenoside Rb1. Compared with that in the Normal group, FAP activity was lower in the Control group. The viability of FAPs was increased after the addition of F10 medium containing different concentrations of ginsenoside Rb1. Among them, 60 µM ginsenoside Rb1 had the greatest effect on FAPs (Fig. 1B). In addition, the Collagen II, FN and α-SMA mRNA and protein levels in the Control group were increased compared with those in the Normal group. Collagen II, FN and α-SMA mRNA and protein levels decreased after treatment with different concentrations of ginsenoside Rb1 in F10 medium. Among them, 60 µM ginsenoside Rb1 had the greatest effect on these proteins (Fig. 1C and D). Therefore, 60 µM ginsenoside Rb1 was selected for subsequent study.

### Ginsenoside Rb1 improved RCTs in rats

**Fig. 2:**
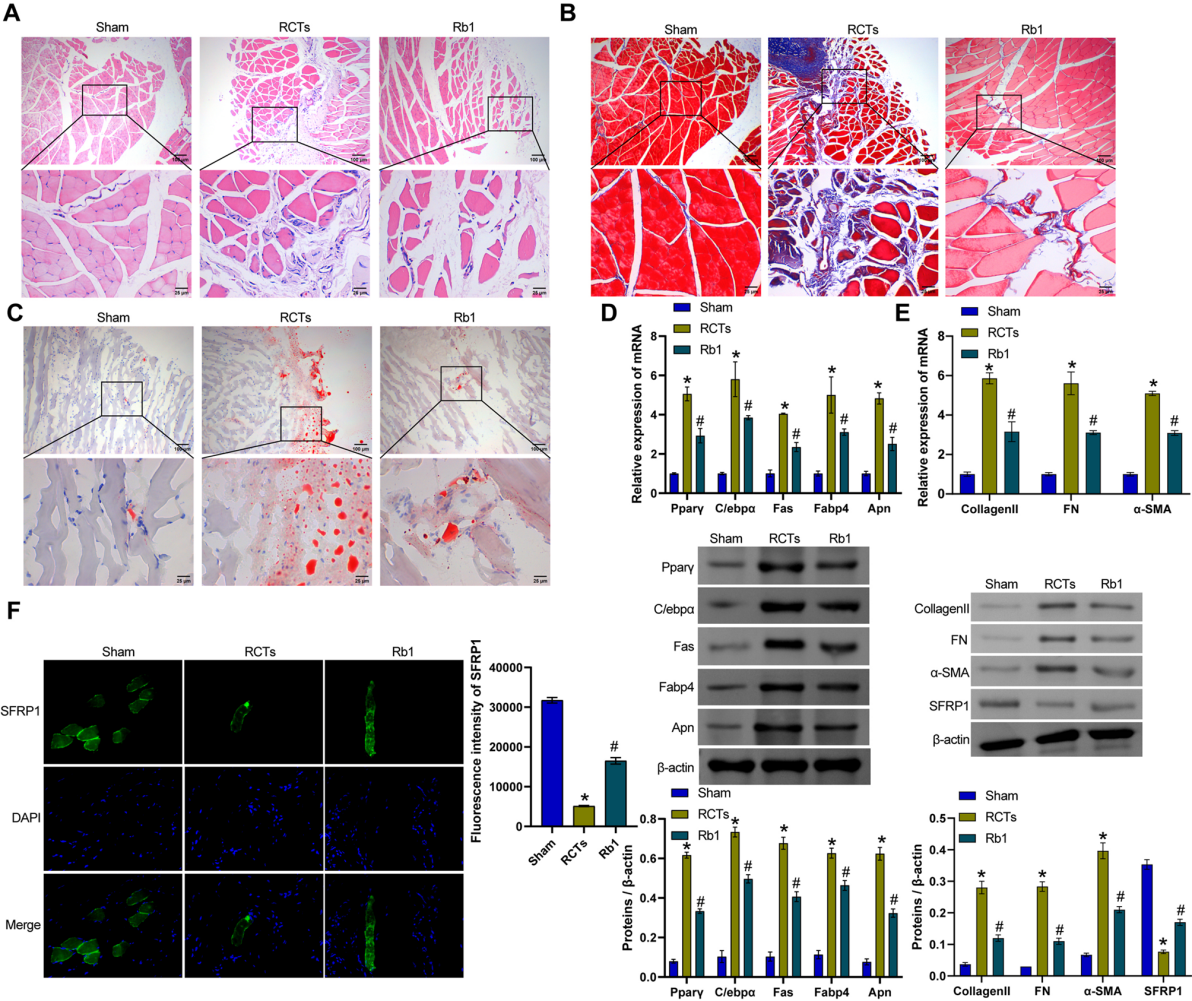
Ginsenoside Rb1 improved RCTs in rats. **A**. HE staining of the injured tissue from each group. **B**. Masson’s trichrome staining of infiltrated tissue fibers. **C**. Oil red O staining of fat accumulation in the tissue. **D**. qRT‒PCR and Western blot were performed to detect the expression of lipid-related molecules (Pparγ, C/ebpα, Fas, Fabp4, and Apn). **E**. Collagen II, FN and α-SMA mRNA levels. **F**. Protein expression of Collagen II, FN, α-SMA and SFRP1. **G**. IF staining showing SFRP1 levels. *P < 0.05 compared with the Sham group and #*P* < 0.05 compared with the RCTs group

Next, we established a rat model of RCTs and treated them with ginsenoside Rb1. HE staining revealed that the cells in the Sham group were arranged neatly and evenly. The RCTs group exhibited cell infiltration and a tissue cavity. The cell infiltration in the Rb1 group improved (Fig. 2A). Masson staining revealed that tendon fiber infiltration in the RCTs group was observably increased compared with the Sham group. The infiltration was alleviated by treatment with ginsenoside Rb1 (Fig. 2B). Oil red O staining revealed greater fat accumulation in the tendons of the RCTs group than in those of the Sham group. After the use of ginsenoside Rb1, fat accumulation was reduced (Fig. 2C). Next, qRT‒PCR and Western blot were performed to detect the expression of lipid-related molecules (Pparγ, C/ebpα, Fas, Fabp4, and Apn). Compared with the Sham group, the expression of Pparγ, C/ebpα, Fas, Fabp4, and Apn was increased in the RCTs group. After the administration of ginsenoside Rb1, the expression of Pparγ, C/ebpα, Fas, Fabp4, and Apn was reduced (Fig. 2D). In addition, compared with the Sham group, Collagen II, FN and α-SMA mRNA and protein levels were increased in the RCTs group, whereas SFRP1 protein expression was decreased. After the administration of ginsenoside Rb1, the mRNA and protein levels of Collagen II, FN and α-SMA decreased, whereas SFRP1 protein expression increased (Fig. 2E to G). Our results suggested that ginsenoside Rb1 improved RCTs in rats.

### Ginsenoside Rb1 targeted SFRP1

**Fig. 3:**
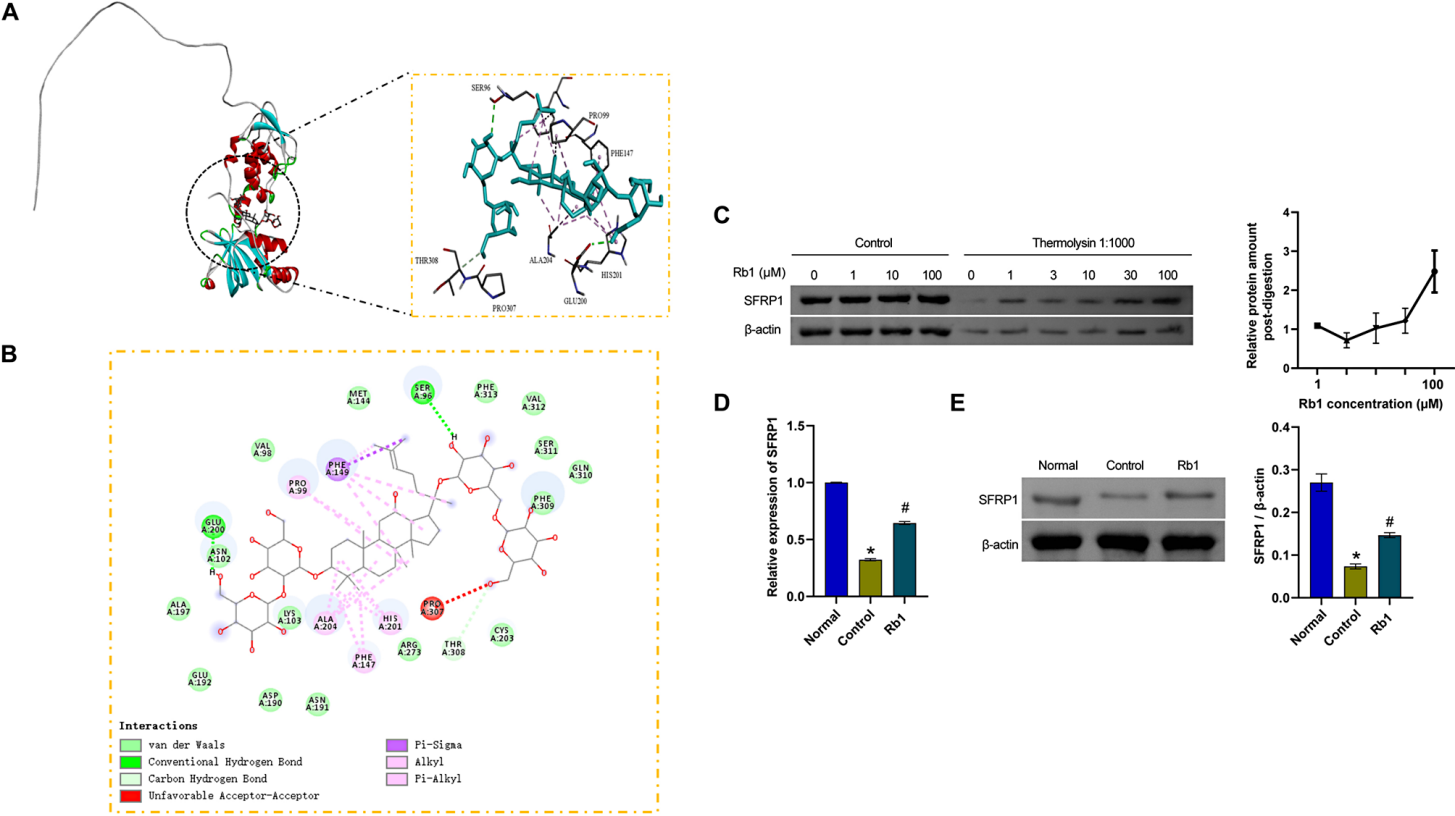
Ginsenoside Rb1 targeted SFRP1. **A**. Structural analysis of the main active components of ginsenoside Rb1. **B**. Molecular docking analysis of ginsenoside Rb1 binding to SFRP1. **C**. The DARTS assay was used to investigate the interaction between ginsenoside Rb1 and SFRP1. **D** and **E**. SFRP1 mRNA and protein expression. *P < 0.05 compared with the Normal group and #*P* < 0.05 compared with the Control group

Next, as shown in Fig. 3A, we performed a structural analysis of the main active components of ginsenoside Rb1. Molecular docking revealed the binding of ginsenoside Rb1 to SFRP1. The binding energy of this docking was − 7.9 kcal/mol, with a value less than − 4.0 kcal/mol, suggesting that the compound bound to the protein spontaneously. As shown in Fig. 3B, the compounds interacted with the protein mainly through hydrogen bonding interactions and hydrophobic interactions. The compound formed stable hydrogen bonds with GLU 200, SER 96, and THR 308. The hydrophobic groups in the compound formed hydrophobic interactions with amino acids such as ALA 204, HIS 201, PHE 147, PHE 149, and PRO 99 in the protein A chain, which were the main forces that promoted the binding of the compound to the active site. The DARTS assay revealed that ginsenoside Rb1 protected the SFRP1 protein from digestion by high-concentration thermolysin (1:1000) in a dose-dependent manner, indicating that ginsenoside Rb1 had a targeting relationship with the SFRP1 protein (Fig. 3C). Additionally, the SFRP1 levels in the Control group were lower than those in the Normal group. After ginsenoside Rb1 treatment in F10 medium, SFRP1 levels increased (Fig. 3D and E). Our results suggested that ginsenoside Rb1 could target SFRP1.

### Ginsenoside Rb1 improved RCTs by influencing SFRP1 to inhibit fat deposition and fibrosis

**Fig. 4:**
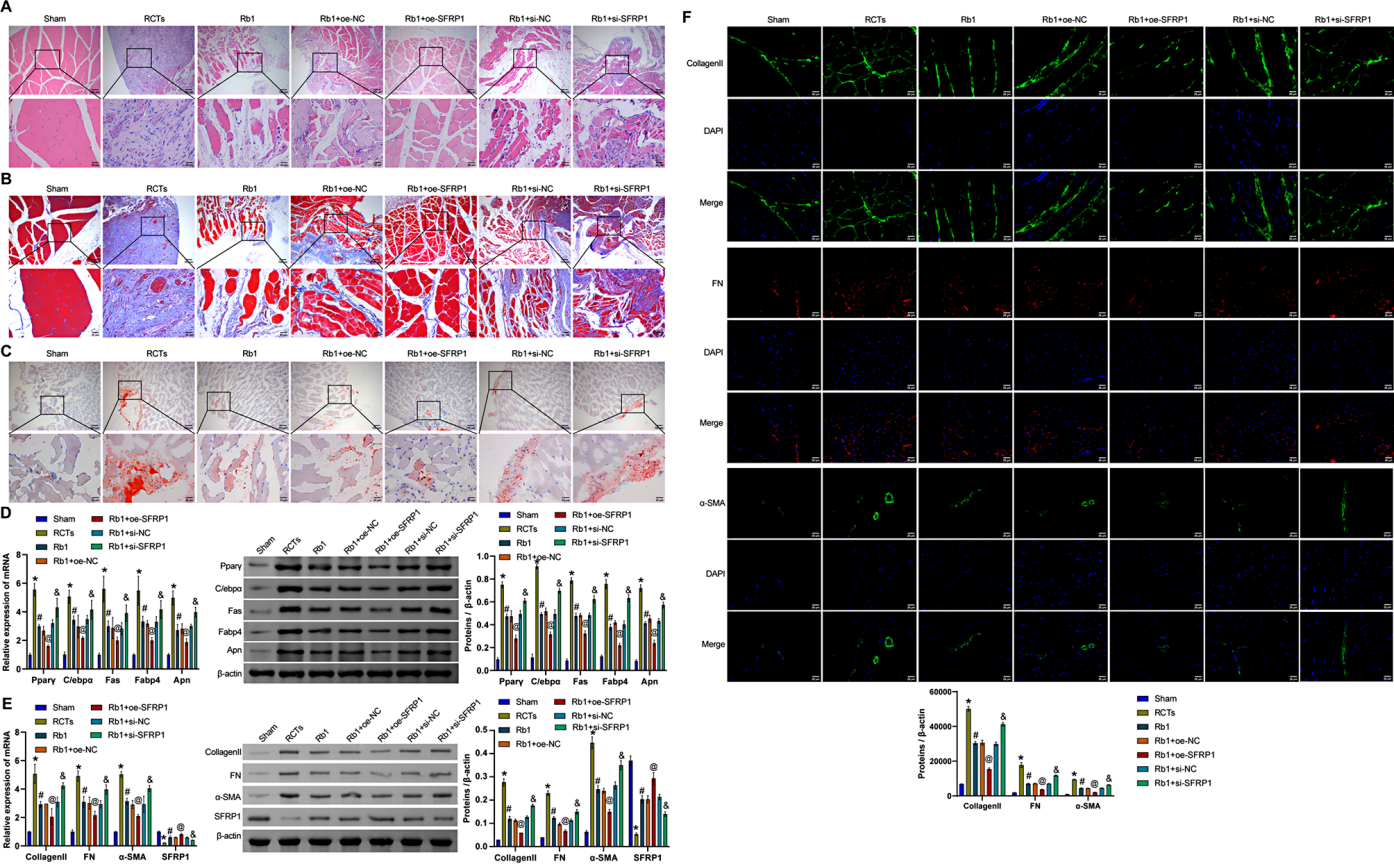
Ginsenoside Rb1 improved RCTs by influencing SFRP1 to inhibit fat deposition and fibrosis. **A**. HE staining of the injured tissue from each group. **B**. Masson staining of infiltrated tissue fibers. **C**. Oil red O staining of fat accumulation in the tissue. **D**. qRT‒PCR and Western blot were utilized to measure the expression of lipid-related molecules (Pparγ, C/ebpα, Fas, Fabp4, and Apn). **E**. Collagen II, FN, α-SMA and SFRP1 mRNA and protein expression. **F**. IF staining showing Collagen II, FN, and α-SMA levels. *P < 0.05 compared with the Sham group, #*P* < 0.05 compared with the RCTs group, @*P* < 0.05 compared with the Rb1 + oe-NC group, and &*P* < 0.05 compared with the Rb1 + si-NC group

In addition, SFRP1 was knocked down and overexpressed in vivo. HE staining revealed that the cells in the Sham group were arranged neatly and evenly. The RCTs group had cell infiltration and a tissue cavity. The Rb1 group showed improvement in cell infiltration. After the overexpression of SFRP1 and ginsenoside Rb1 treatment, cell infiltration was further alleviated. However, after further knocking down SFRP1, cell infiltration increased (Fig. 4A). Masson staining revealed that tendon fiber infiltration in the RCTs group was observably increased compared with that in the Sham group. The infiltration was alleviated by treatment with ginsenoside Rb1. After the overexpression of SFRP1 and ginsenoside Rb1 treatment, the fibrous infiltration of tendon tissue was further relieved. However, after further knockdown of SFRP1, the fiber infiltration of tendon tissue was aggravated (Fig. 4B). Oil red O staining revealed greater fat accumulation in the tendons of the RCTs group than in those of the Sham group. After the administration of ginsenoside Rb1, fat accumulation was reduced. After SFRP1 overexpression and ginsenoside Rb1 treatment, the accumulation of fat in the tendon tissue was further alleviated. However, after further interference with SFRP1, the accumulation of fat in tendon tissue increased (Fig. 4C). Then, qRT‒PCR and Western blot were performed to detect the expression of lipid-related molecules (Pparγ, C/ebpα, Fas, Fabp4, and Apn). Compared with the Sham group, Pparγ, C/ebpα, Fas, Fabp4, and Apn levels were increased in the RCTs group. After the administration of ginsenoside Rb1, Pparγ, C/ebpα, Fas, Fabp4, and Apn levels decreased. After the overexpression of SFRP1 and treatment with ginsenoside Rb1, Pparγ, C/ebpα, Fas, Fabp4, and Apn levels were further decreased. However, after further knockdown of SFRP1, Pparγ, C/ebpα, Fas, Fabp4, and Apn levels increased (Fig. 4D). In addition, compared with the Sham group, the Collagen II, FN and α-SMA levels were increased in the RCTs group, whereas the SFRP1 level was decreased. After the administration of ginsenoside Rb1, the Collagen II, FN and α-SMA levels decreased, whereas the SFRP1 level increased. After the overexpression of SFRP1 and treatment with ginsenoside Rb1, the Collagen II, FN and α-SMA levels were further decreased, whereas the SFRP1 level was further increased. However, after the knockdown of SFRP1, the Collagen II, FN and α-SMA levels increased, whereas the SFRP1 level decreased (Fig. 4E and F). Our results suggested that ginsenoside Rb1 could improve RCTs by influencing SFRP1 to inhibit fat deposition and fibrosis.

### Ginsenoside Rb1 affected mitochondrial Ca2+ transport by regulating the wnt signaling pathway through SFRP1

**Fig. 5:**
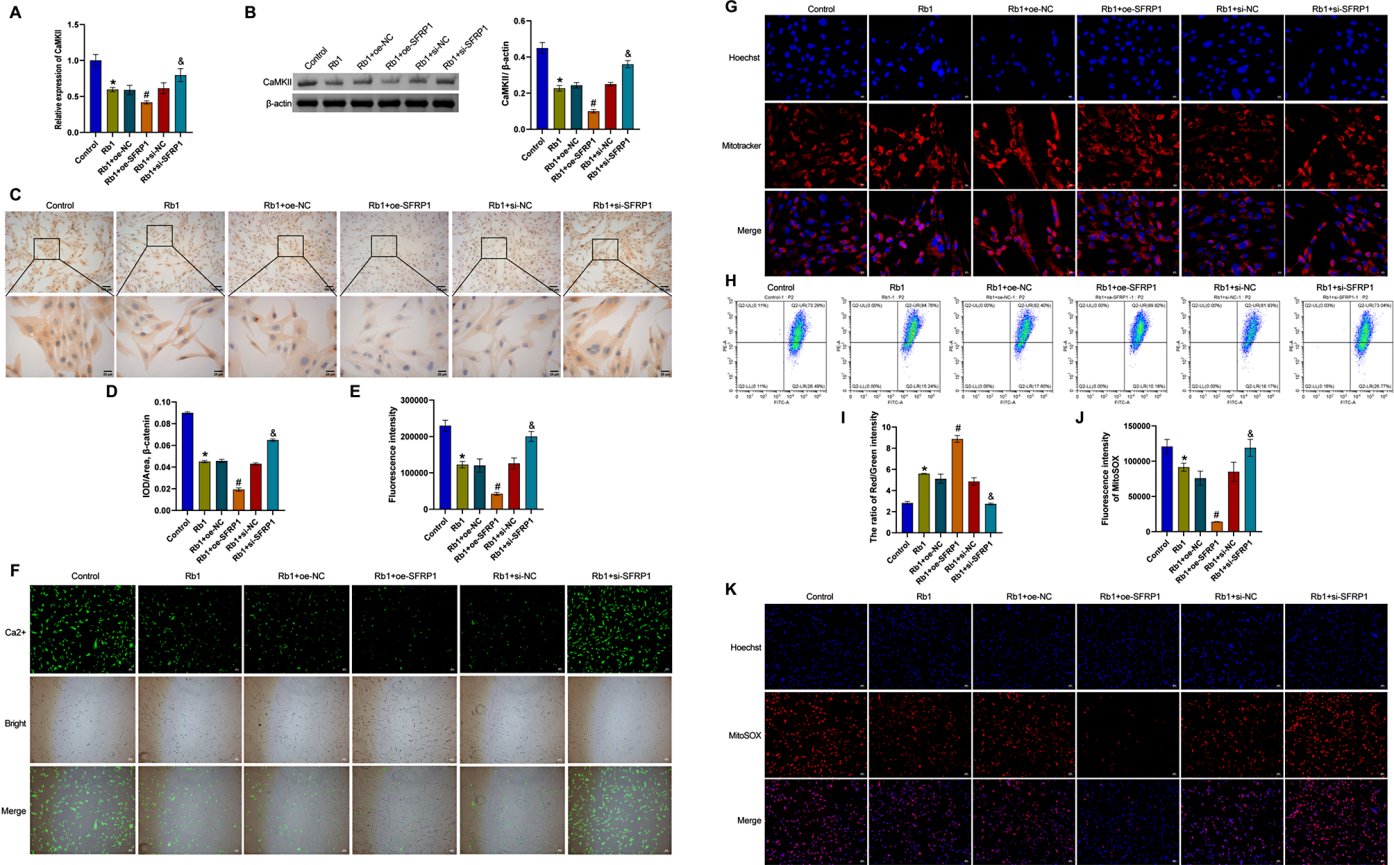
Ginsenoside Rb1 affected mitochondrial Ca^2+^ transport by regulating the Wnt signaling pathway through SFRP1. **A** and **B**. CaMKII mRNA and protein expression. **C** and **D**. ICC analysis of the distribution of β-catenin. **E** and **F**. Changes in Ca^2+^ absorption were detected via fluorescence calcium imaging. **G**. MitoTracker Red CMXRos staining of mitochondrial morphology. **H** and **I**. The mitochondrial membrane potential was evaluated with a JC-1 kit. **J** and **K**. MitoSOX staining was utilized to measure mitochondrial ROS. *P < 0.05 compared with the Control group, #*P* < 0.05 compared with the Rb1 + oe-NC group, and &*P* < 0.05 compared with the Rb1 + si-NC group

SFRP1 was knocked down and overexpressed in vitro. Compared with the Control group, the CaMKII and β-catenin levels were decreased in the Rb1 group. After SFRP1 overexpression and ginsenoside Rb1 treatment, CaMKII and β-catenin levels were further decreased. After SFRP1 knockdown, CaMKII and β-catenin levels were elevated (Fig. 5A to D). Moreover, compared with the Control group, the Rb1 group presented a decrease in the Ca^2+^ fluorescence intensity, a reduction in the mitochondrial length, an increase in the red/green intensity, and a decrease in MitoSOX fluorescence intensity. After SFRP1 overexpression and ginsenoside Rb1 treatment, the Ca^2+^ fluorescence intensity was further decreased, the mitochondrial length was further decreased, the red/green intensity was further increased, and the MitoSOX fluorescence intensity was further decreased. However, after SFRP1 was knocked down, the Ca^2+^ fluorescence intensity increased, the mitochondrial length increased, the red/green intensity decreased, and the MitoSOX fluorescence intensity increased (Fig. 5E to K). Our results suggested that ginsenoside Rb1 could influence mitochondrial Ca^2+^ transport by regulating the Wnt signaling pathway through SFRP1.

### Ginsenoside Rb1 regulated wnt signaling pathway through SFRP1 to affect mitochondrial Ca2+ transport and inhibit cell damage and fibrosis

**Fig. 6:**
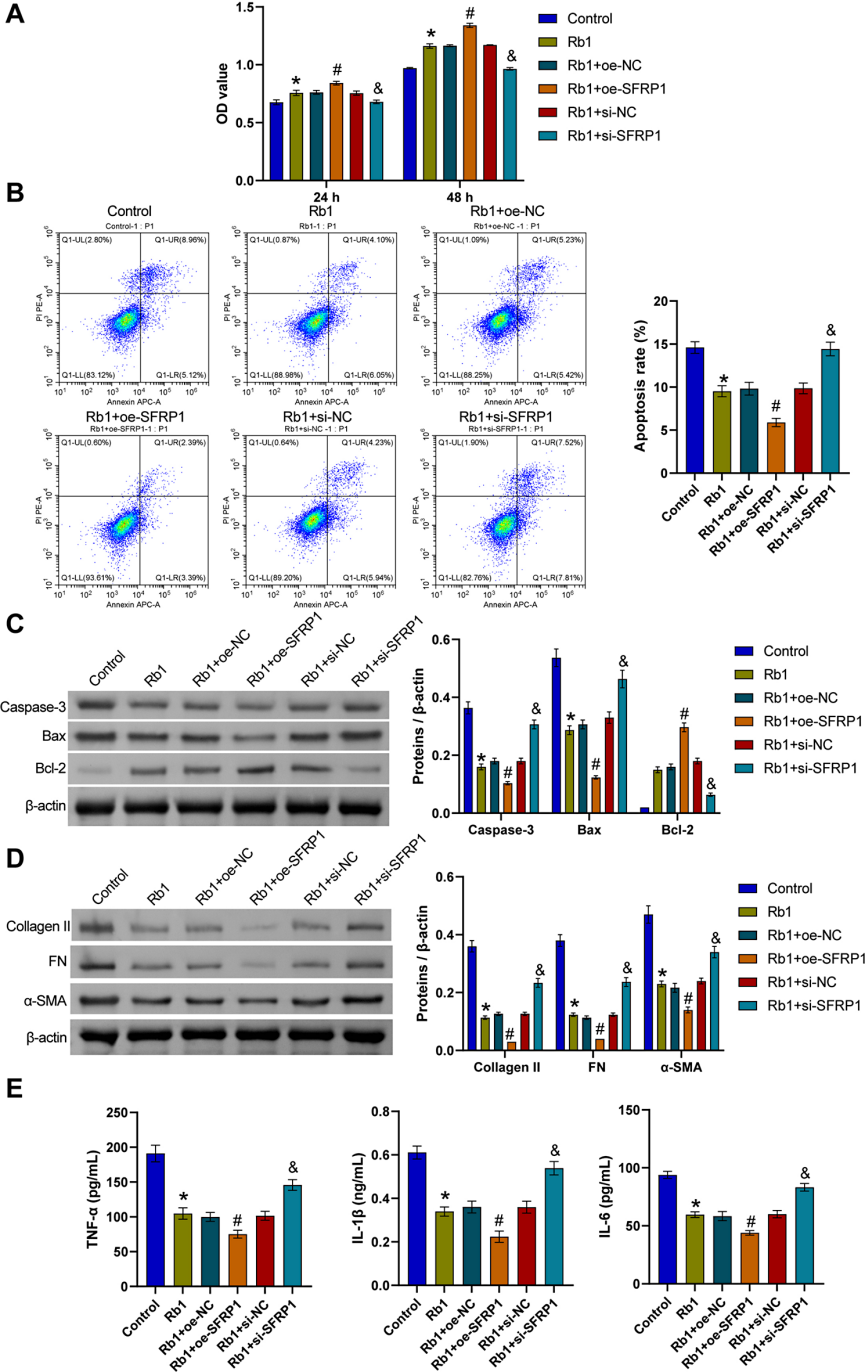
Ginsenoside Rb1 regulated the Wnt signaling pathway through SFRP1 to affect mitochondrial Ca^2+^ transport and inhibit cell damage and fibrosis. **A**. The CCK-8 assay was performed to detect cell proliferation in each group. **B**. Apoptosis was determined by flow cytometry. **C**. Western blot analysis of Caspase-3, Bax, and Bcl-2 levels. **D**. Western blot analysis of Collagen II, FN and α-SMA levels in cells. **E**. The contents of TNF-α, IL-1β and IL-6. *P < 0.05 compared with the Control group, #*P* < 0.05 compared with the Rb1 + oe-NC group, and &*P* < 0.05 compared with the Rb1 + si-NC group

**Fig. 7:**
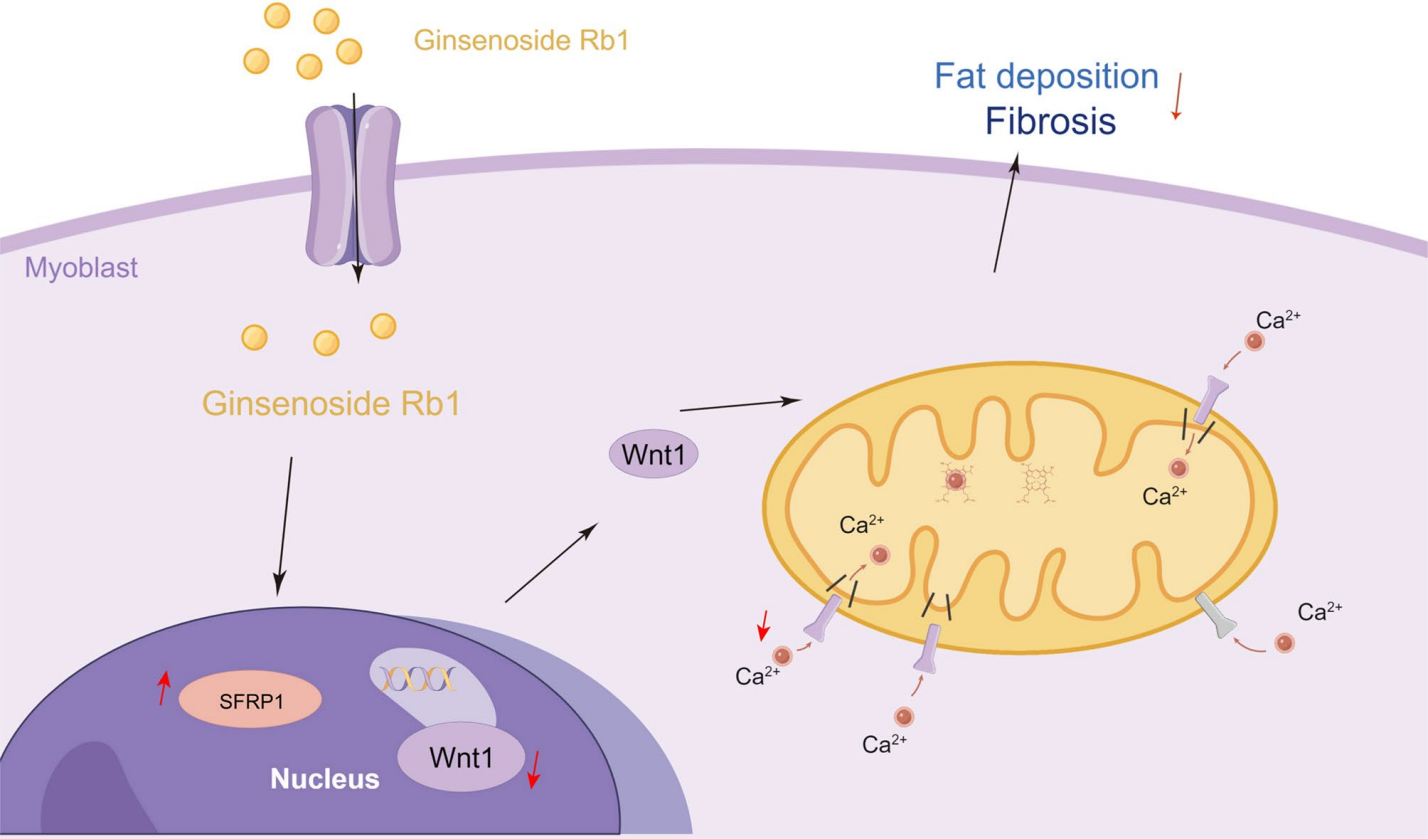
Graphical abstract. The mechanism by which ginsenoside Rb1 treats fat infiltration and muscle fibrosis caused by RCTs

Compared with the Control group, cell proliferation was increased, apoptosis was decreased, Bcl-2 levels were increased, and Caspase-3 and Bax levels were decreased in the Rb1 group. After the overexpression of SFRP1 and treatment with ginsenoside Rb1, cell proliferation was further increased, apoptosis was further decreased, the Bcl-2 level was further increased, and the levels of Caspase-3 and Bax were further decreased. However, after the knockdown of SFRP1, cell proliferation decreased, apoptosis increased, the level of Bcl-2 decreased, and Caspase-3 and Bax levels increased (Fig. 6A to C). In addition, the Collagen II, FN and α-SMA levels in muscle tissue were decreased in the Rb1 group compared with the Control group, and the TNF-α, IL-1β and IL-6 levels were decreased. After the overexpression of SFRP1 and treatment with ginsenoside Rb1, the Collagen II, FN and α-SMA levels in muscle tissue were further reduced, and the TNF-α, IL-1β and IL-6 contents were further reduced. However, after the knockdown of SFRP1, the Collagen II, FN and α-SMA levels in the cells increased, and the TNF-α, IL-1β and IL-6 levels increased (Fig. 6D and E). Our results suggested that ginsenoside Rb1 could regulate the Wnt signaling pathway through SFRP1 to affect mitochondrial Ca^2+^ transport and inhibit cell damage and fibrosis. Furthermore, Fig. 7 showed that ginsenoside Rb1 could inhibit the activation of the Wnt signaling pathway by promoting SFRP1 expression, thereby inhibiting mitochondrial function and Ca^2+^ absorption to fat infiltration and muscle fibrosis caused by RCTs.

## Discussion

RCTs seriously affect shoulder mobility in patients (Liu et al. 2022) and may be associated with pain, weakness, and shoulder dysfunction (Eckers et al. 2023). The treatment of RCTs should be individualized according to the patient’s age, needs, and symptoms (St Pierre 2024). In this study, we investigated the mechanism of ginsenoside Rb1 in RCTs through in vivo and in vitro experiments. We found that ginsenoside Rb1 could inhibit the activation of the Wnt signaling pathway by promoting SFRP1 expression, thereby inhibiting mitochondrial function and Ca^2+^ absorption to treat fat infiltration and muscle fibrosis caused by RCTs. This study is also the first to report the effect of ginsenoside Rb1 on SFRP1 to regulate the Wnt signaling pathway in an RCTs model.

Ginsenoside Rb1 is a typical component of ginseng and has anti-inflammatory, antioxidant, antiapoptotic and antiautophagy activities in the nervous system (Ling et al. 2024). During cerebral ischemia, ginsenoside Rb1 inhibits NADH dehydrogenase in mitochondrial complex I and blocks ROS produced by reverse electron transport in complex I, thus inactivating astrocytes and protecting mitochondria (Ni et al. 2022a, b). In addition, ginsenoside Rb1 alleviates myocardial ischemia/reperfusion injury by repressing mitochondrial complex I-mediated production of ROS (Jiang et al. 2021). In aldose reductase-overexpressing podocytes, ginsenoside Rb1 inhibits aldose reductase-mediated ROS overproduction and protects against high glucose-induced mitochondrial damage (He et al. 2022). Moreover, ginsenoside Rb1 can reduce plasma transaminase activity and liver inflammation and inhibit liver fibrosis (Hou et al. 2014). In this study, we found that FAP viability was lower in the Control group than in the Normal group and that the Collagen II, FN and α-SMA mRNA and protein levels were increased. After treatment with different concentrations of ginsenoside Rb1, FAP viability increased, and Collagen II, FN and α-SMA levels decreased. Among the concentrations tested, 60 µM ginsenoside Rb1-treated F10 medium had the greatest effect. In vivo studies subsequently showed that ginsenoside Rb1 improved fat infiltration and muscle fibrosis in rats in RCTs. Therefore, we explored the specific mechanism by which ginsenoside Rb1 improved RCTs.

SFRPs are a family of secreted proteins that bind to Wnt ligands and curl receptors, thereby regulating the Wnt signaling cascade (Esteve and Bovolenta 2010). SFRP1 is a mature inhibitor of the Wnt signaling pathway, and its polymorphisms are associated with risks of inflammation, infection and cancer (Zhao et al. 2016). SFRP1 deletion or silencing involves epigenetic mechanisms and is involved in biological behaviors such as cell proliferation, migration, and pyroptosis, leading to disease progression and a poor prognosis (Jiang et al. 2022). The tumor suppressor gene SFRP1 is downregulated in tumor tissues (Bernichtein et al. 2009). However, the role of SFRP1 in RCTs is unclear. In this study, we observed the binding of ginsenoside Rb1 to SFRP1 via molecular docking. Additionally, the SFRP1 levels in the Control group were lower than those in the Normal group. After treatment with ginsenoside Rb1, SFRP1 levels increased. Further in vivo experiments revealed that ginsenoside Rb1 could treat RCTs by influencing SFRP1 to inhibit fat deposition and fibrosis. Therefore, we further explored the mechanism by which Rb1 docks to SFRP1 in RCTs.

Wnt signaling plays an essential role not only in embryonic development and morphogenesis but also in the pathogenesis of various diseases, including cancer (Ohnaka 2006). Studies have shown that Wnt/β-catenin signaling plays a role in abnormal wound repair and fiber formation (Lam and Gottardi 2011). The overexpression of SIRT6 in rotator cuff and primary tendon cells has been reported to modulate typical Wnt signaling via the repression of the transcription of sclerosin, a Wnt antagonist (Moon et al. 2023). Mitochondria are essential for life and provide biological energy for other organelles and cellular physiological processes (Wang et al. 2021). Mitochondrial matrix Ca^2+^ is a key regulator of ATP production and cell death (Murphy and Steenbergen 2021). In this study, ginsenoside Rb1 affected mitochondrial Ca^2+^ transport in vitro by regulating the Wnt signaling pathway through SFRP1. In addition, ginsenoside Rb1 regulated the Wnt signaling pathway through SFRP1 to affect mitochondrial Ca^2+^ transport and inhibit cell damage and fibrosis. Our study confirms that ginsenoside Rb1 regulates the Wnt signaling pathway through SFRP1 by influencing mitochondrial Ca^2+^ transport and inhibiting fat deposition and fibrosis in RCTs.

Currently, relatively little research has been conducted on ginsenoside Rb1 and its association with RCTs, with a focus on its impacts on cell proliferation, differentiation, and inflammatory responses. The Wnt signaling pathway is one of the more extensively studied pathways in this context. However, when investigating its potential therapeutic role in RCTs, in addition to the Wnt signaling pathway, other biological pathways may also be involved. Research has shown that the TGF-β/bone morphogenetic protein (BMP) pathway is one of the most important and complex signaling systems in vascular development (Cunha et al. 2017) and is involved in cell proliferation and differentiation (Guo et al. 2017). Furthermore, the MAPK signaling pathway, NF-κB signaling pathway, cAMP signaling pathway, and other pathways may be involved in the regulation of RCTs (Ren et al. 2022). Therefore, ginsenoside Rb1 may play a role in RCTs through the TGF-β/BMP pathway, the MAPK signaling pathway, the NF-κB signaling pathway, the cAMP signaling pathway, and other pathways. In future research, we will explore the effects of ginsenoside Rb1 on other signaling pathways to further elucidate its mechanism of action. We believe that by incorporating discussions on other signaling pathways and considering them in future research, our study will become more in-depth and comprehensive. We look forward to making a greater contribution to the scientific development of this field through broader research endeavors.

Existing treatments for RCTs typically include enhancing muscle strength and range of motion through specific exercises and therapeutic programs (Ainsworth 2006), using nonsteroidal anti-inflammatory drugs to alleviate pain and inflammation (Sewpaul et al. 2024), and injecting corticosteroids to reduce inflammation (García et al. 2024). In severe cases, surgery may be required to repair the torn tendons (Karjalainen et al. 2019). The potential distinction between ginsenoside Rb1 treatment for RCTs and existing methods is that ginsenoside Rb1 may promote tendon repair by promoting the proliferation and differentiation of tendon cells and collagen synthesis rather than just relieving symptoms. Additionally, ginsenoside Rb1 may exert anti-inflammatory effects, mitigating inflammatory responses by modulating the release of inflammatory mediators. As a natural product, ginsenoside Rb1 may offer better safety and tolerability, especially in long-term treatment. Ginsenoside Rb1 might be administered orally, eliminating the need for injections or surgery. However, currently, a relative scarcity of research is available on the use of ginsenoside Rb1 for treating RCTs, and more clinical studies are needed to verify its efficacy and safety. We plan to design and conduct randomized controlled trials in the future to assess the practical effectiveness of ginsenoside Rb1 in a clinical setting.

Differences exist between animal models and human diseases; hence, preclinical studies are needed to predict clinical outcomes. Although our experimental results are encouraging, the clinical application potential of ginsenoside Rb1 needs to be assessed through well-designed clinical trials. In terms of clinical trials, we also need to evaluate the safety of ginsenoside Rb1, including short-term and long-term toxicity studies, drug interactions, and monitoring potential adverse reactions. In the future, we will conduct larger-scale clinical trials and explore the possibility of using ginsenoside Rb1 in combination with other therapeutic approaches.

## Conclusions

In this study, we evaluated the mechanism of ginsenoside Rb1 in RCTs and identified that its potential mechanism of action was related to the Wnt signaling pathway and mitochondrial Ca^2+^ transport. Our results suggest a new way for ginsenoside Rb1 to improve RCTs. In addition, a better understanding of the roles of the Wnt signaling pathway and mitochondrial Ca^2+^ transport in RCTs may provide a theoretical basis for developing innovative treatment options for RCTs.

## Electronic supplementary material

Below is the link to the electronic supplementary material.

Supplementary Material 1

## Data Availability

The data supporting the conclusion are included in the article.
