# Supplementary figures and images for: Ginsenoside Rb1 affects mitochondrial Ca2+ transport and inhibits fat deposition and fibrosis by regulating the wnt signaling pathway to treat rotator cuff tears via docking with SFRP1
Source: Mol Med. 2024 Dec 2;30:240. doi: 10.1186/s10020-024-01009-0 (PMC11610219; doi:10.1186/s10020-024-01009-0)

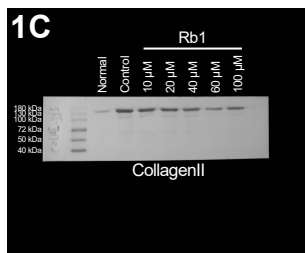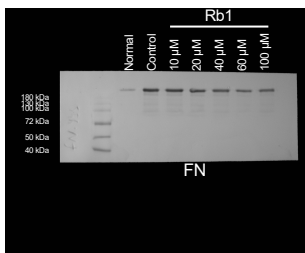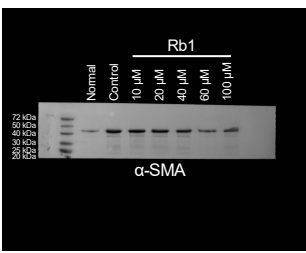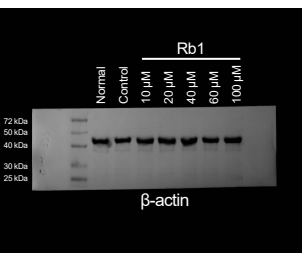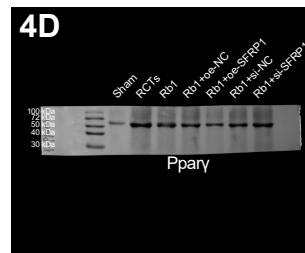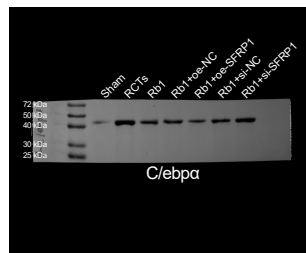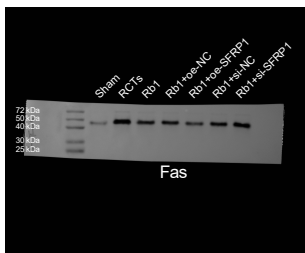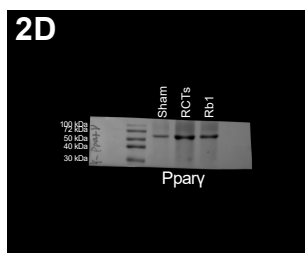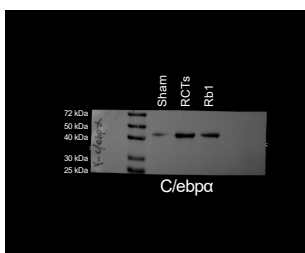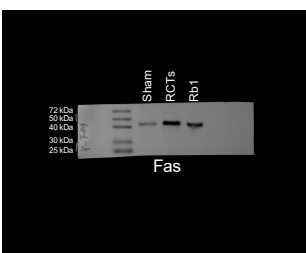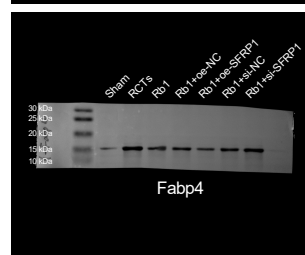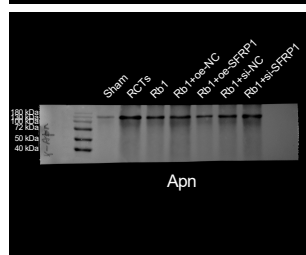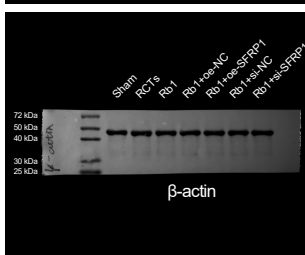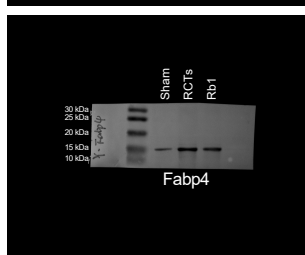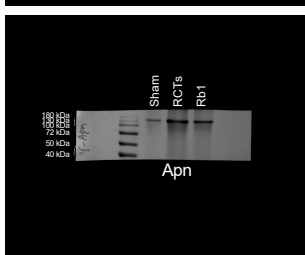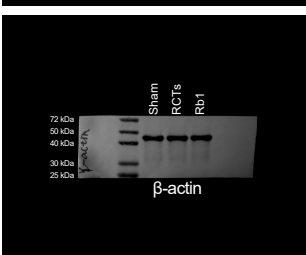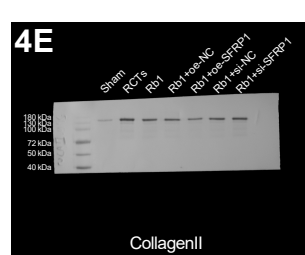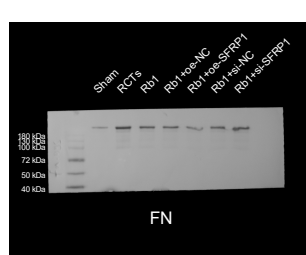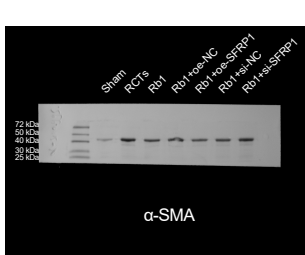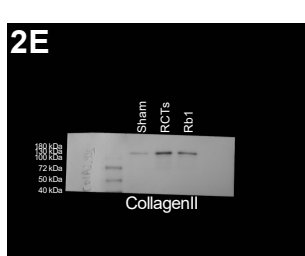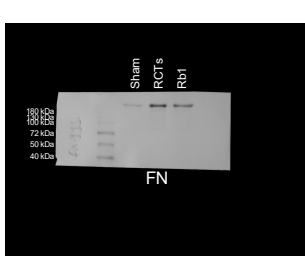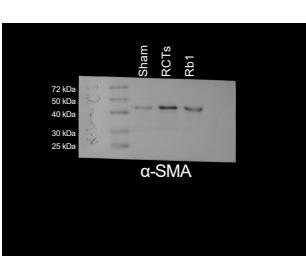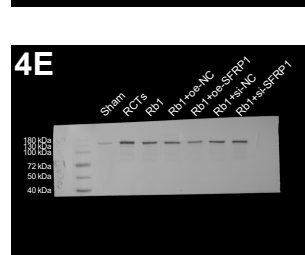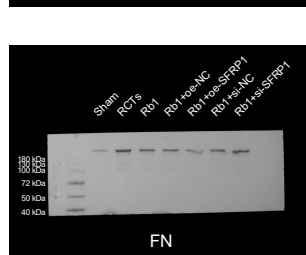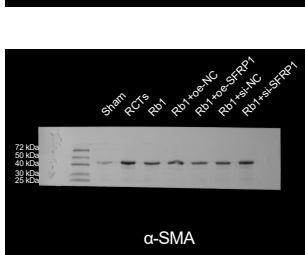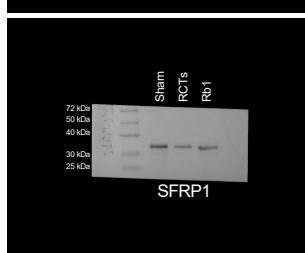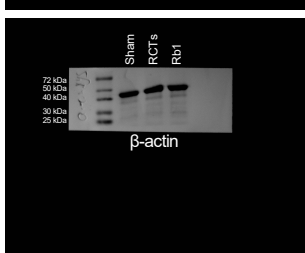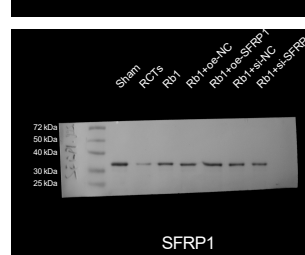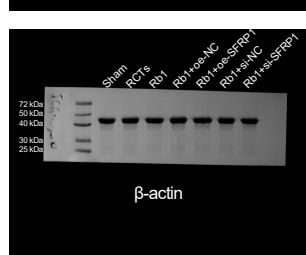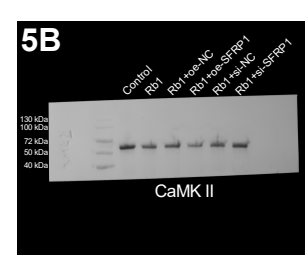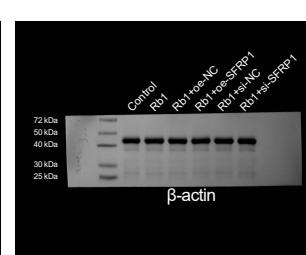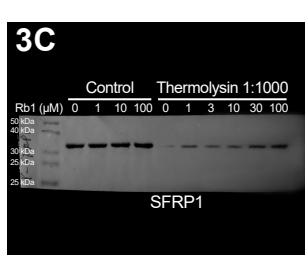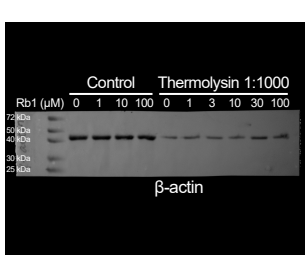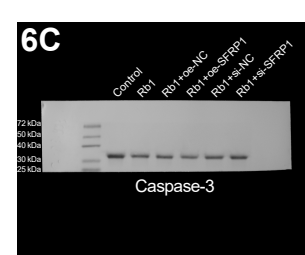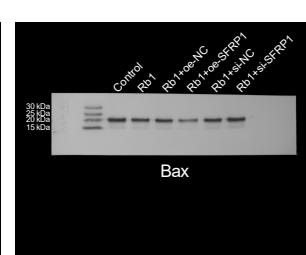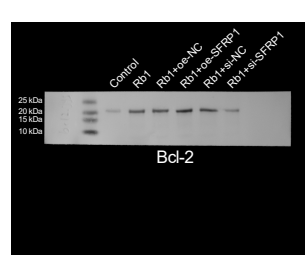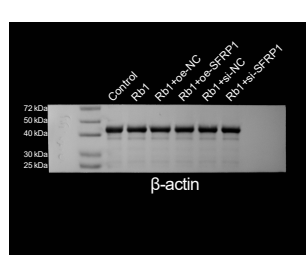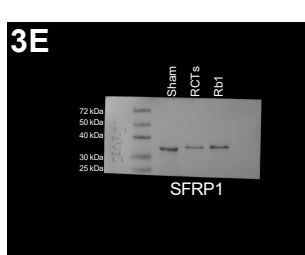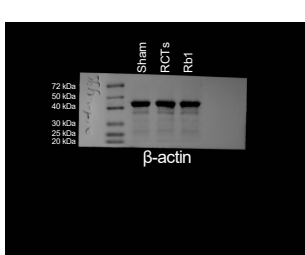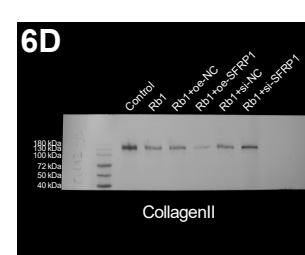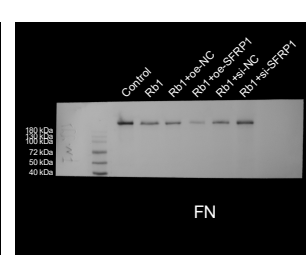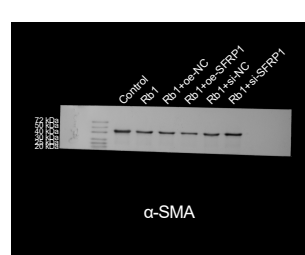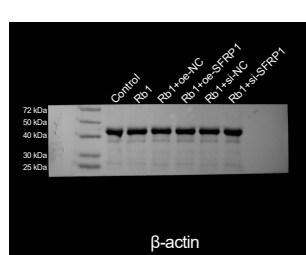

Supplement: Supplementary file 1 — Supplementary Material 1 [file 10020_2024_1009_MOESM1_ESM.pdf]
